# Effect of *TP53* rs1042522 on the susceptibility of patients to oral squamous cell carcinoma and oral leukoplakia: a meta-analysis

**DOI:** 10.1186/s12903-018-0603-6

**Published:** 2018-08-20

**Authors:** Zhen Sun, Wei Gao, Jiang-Tao Cui

**Affiliations:** 1 Department of Stomatology, Second Hospital of Tianjin Medical University, Ping-Jiang Road, He Xi District, 300211 Tianjin, People’s Republic of China; 2 Department of Interventional Therapy, Tianjin Medical University Cancer Institute and Hospital, National Clinical Research Center for Cancer, Key Laboratory of Cancer Prevention and Therapy, Tianjin’s Clinical Research Center for Cancer, Huan Hu West Road, 300060 Tianjin, People’s Republic of China

**Keywords:** TP53, OSCC, OL, Polymorphism, Meta-analysis

## Abstract

**Background:**

There are different and inconsistent conclusions regarding the genetic relationship between the human tumor suppressor p53 (*TP53*) rs1042522 polymorphism and the risk of oral squamous cell carcinoma (OSCC) and oral leukoplakia (OL). Therefore, the aim of the study was to comprehensively reassess this association through the performance of an updated meta-analysis.

**Methods:**

After searching the available databases, we systematically screened and included the eligible case-control studies, which contain the full genotype frequency data of the *TP53* rs1042522 polymorphism for both OSCC/OL patients and the negative control groups. *P*_A_ (*P-*value of the association test) and ORs (odd ratios) with their corresponding 95% CIs (confidence intervals) were calculated to quantitatively evaluate the influence of *TP53* rs1042522 on the susceptibility of patients to OSCC or OL.

**Results:**

In total, twenty eligible case-control articles were finally enrolled. Compared with the controls, no increased or decreased risk of OSCC was observed in the cases for six genetic models including allele C vs. G (*P*_*A*_ = 0.741), carrier C vs. G (*P*_*A*_ = 0.853), homozygote CC vs. GG (*P*_*A*_ = 0.085), heterozygote GC vs. GG (*P*_*A*_ = 0.882), dominant GC + CC vs. GG (*P*_*A*_ = 0.969), and recessive CC vs. GG + GC (*P*_*A*_ = 0.980). Furthermore, no statistically significant difference between the cases and controls was detected in most subgroup meta-analyses (*P*_*A*_ > 0.05). For the risk of OL, we did not observe the difference between the cases and controls for most genetic models in the overall meta-analysis and subsequent subgroup analysis (*P*_*A*_ > 0.05). Begg’s test and Egger’s test excluded the large risk of publication bias within the included studies in the meta-analysis of OSCC. The sensitivity analysis indicated the above relatively stable results.

**Conclusions:**

Our updated meta-analysis (based on the current evidence) shows that *TP53* rs1042522 may not confer susceptibility to OSCC. In addition, for the first time, we provided evidence regarding the negative association between *TP53* rs1042522 and OL risk.

**Electronic supplementary material:**

The online version of this article (10.1186/s12903-018-0603-6) contains supplementary material, which is available to authorized users.

## Background

The human tumor suppressor p53 *(TP53)* gene on chromosome 17p13, which is also known as *p53*, was reported to be involved in a group of cell biology events, such as the cell cycle, apoptosis and genomic stability [1, 2]. Some genetic variants of the *TP53* gene were reported to be linked to human carcinogenesis [2, 3]. The rs1042522 G/C, which is a very common polymorphism at exon 4 of the *TP53* gene, results in the alteration at codon 72 between arginine (Arg, R) and proline (Pro, P) and causes the TP53Arg72Pro mutation. This may affect the normal function of the TP53 protein and is implicated in susceptibility to several clinical diseases (e.g., colorectal cancer [4], endometriosis [5] or type 2 diabetes [6]).

Herein, we are interested in exploring the potential role of *TP53* rs1042522 in the risk of oral squamous cell carcinoma (OSCC) or oral leukoplakia (OL). OSCC, which is the main type of oral cancer, originates from squamous cells on the surface of the oral cavity or oropharynx [7, 8]. OL is considered the pre-cancerous lesion with white or gray keratosis on the oral mucosa [8, 9]. Life style (e.g., tobacco smoking, drinking, and chewing), human papillomavirus (HPV) infection, and other functional variants may be implicated in the etiology of OSCC and OL [7–9].

Currently, the association between rs1042522 of the *TP53* gene and OL/OSCC risks has been inconsistently reported among different populations. For instance, *TP53* rs1042522 was reported to be associated with the risk of oral potentially malignant disorders (OPMD), including OL, in Argentine patients [10]. However, the risk of OL was not found in Taiwanese patients [11]. *TP53* rs1042522 may have been linked to an increased risk of OSCC in an Indian population [12, 13]. However, the negative genetic conclusion between *TP53* rs1042522 and OSCC risk in India was also observed in another report [14]. Additionally, the GC genotype of *TP53* rs1042522 may be associated with a reduced risk of OSCC patients in Italy [15]. Therefore, the meta-analysis provides helpful insights into the genetic role of *TP53* rs1042522 in the susceptibility of the patient to OL or OSCC.

Currently, as far as we know, no meta-analysis has been previously published to investigate the relationship between rs1042522 of the *TP53* gene and the predisposition of OL. Regarding the association between *TP53* rs1042522 and OSCC risk, only two previous meta-analyses were published [16, 17]. Given the newly published case-control studies and the utilization of a strict screening strategy and quantitative synthesis, we performed an updated meta-analysis aiming to analyze the potential difference of *TP53* rs1042522 in the OSCC cases and the negative controls.

## Materials and methods

### Database searching

Our study was conducted in accordance with PRISMA (preferred reporting items for systematic reviews and meta-analyses) guidelines [18]. The PRISMA 2009 checklist is shown in the Additional file 1. Three online databases, including PUBMED, WOS (Web of Science), and EMBASE (Excerpta Medica Database), were searched up to June 2018 without any restrictions regarding language or the publication period. The principle of PICOS, namely, “population” (P), “intervention” (I), “comparator” (C), “outcomes” (O), and “study designs” (S), was considered. A series of terms regarding “population” (human patients with OSCC or OL disease) and “intervention” (polymorphism of the *TP53* gene) was utilized. To prevent the excessive filtering of articles, we checked the information of the “comparator” (negative control), “outcomes” (risk of OSCC or OL) and “study designs” (case-control study) by reading the text of the articles without a specific limitation in the electronic database search. The detailed search terms are shown in Additional file 2. Then, we removed the duplicates by using the “Find Duplicates” function of Endnote X7 software (Thomson Reuters, Philadelphia, PA, USA).

### Inclusion and exclusion criteria

With reference to our inclusion and exclusion criteria, two authors (ZS and WG) independently screened and assessed the articles for eligibility based on the PICOS strategy. The inclusion criteria were as follows: (P) containing the patients with oral squamous cell carcinoma and oral leukoplakia; (I) focusing on the *TP53* rs1042522 polymorphism; (C) containing the negative controls; (O) the completed genotype distribution of GG, GC and CC and can be used for the assessment of OSCC or OL risk under the six genetic models, namely, C vs. G (allele), C vs. G (carrier), CC vs. GG (homozygote), GC vs. GG (heterozygote), GC + CC vs. GG (dominant), and CC vs. GG + GC (recessive); and (S) case-control studies.

The exclusion criteria were as follows: (P) animal or cell data, other disease or unconfirmed OSCC; (I) other genes, other variants or unconfirmed *TP53* mutation site; (C) lack of a control group or the genotype distributions of the control deviated from the HWE (Hardy-Weinberg Equilibrium) (*P*-value of HWE from χ^2^ test < 0.05); (O) lack of full genotype frequency data in both the case and control group; and (S) a meta-analysis, review, and meeting abstracts.

### Data collection and quality assessment

Then, we carefully extracted the data and listed the basic information (such as method, age, gender, smoking, alcohol, location, ethnicity, and disease type) and genotype frequency in the Tables. E-mails were sent for the missing data. We also evaluated the quality of each study using the NOS (Newcastle-Ottawa quality assessment Scale) system with the score of 1~ 9. The high quality was considered when the NOS score was larger than five. A full discussion was required for a conflicting or controversial issue during quality assessment.

### Association and heterogeneity test

STATA 12.0 software (Stata Corporation, Texas, USA) was used for the quantitative synthesis and outcome measures. A two-sided *P*-value of association test, pooled ORs (odd ratios), and the 95% CI (confidence interval) were performed and used under the following six genetic models: C vs. G (allele); C vs. G (carrier); CC vs. GG (homozygote); GC vs. GG (heterozygote); GC + CC vs. GG (dominant); and CC vs. GG + GC (recessive). When *P* < 0.05 from the association test and the OR value > 1, the C minor allele of *TP53* rs1042522 will be considered the risk factor of OSCC or OL.

We performed the Q statistic and I^2^ test to assess the between study heterogeneity. A random-effect model (DerSimonian and Laird method) for high heterogeneity will be used when *P*-values of the Q statistic are < 0.05 or the I^2^ values are > 50%. Otherwise, a fixed-effect model (Mantel-Haenszel method) was used.

Additionally, we performed a group of subgroup analyses based on the control source (population-based or hospital-based), ethnicity (Caucasian or Asian), location (India, USA, China), and OSCC type (oral cavity, HPV16 −/+).

### Publication bias

Taking into consideration that publication bias may exist, Begg’s test and Egger’s test were conducted when at least ten case-control studies were enrolled. Publication bias was indicated by a *P* value for Begg’s test and Egger’s test being less than 0.05.

### Sensitivity analysis

We also conducted the sensitivity analyses under all of the genetic models. If there is no obvious change for the value of recalculated ORs (odd ratios), and the 95% CI (confidence interval) when the individual study was systematically omitted at a time, statistical stability of data was considered. The deleted case-control studies, which lead to an obvious change, will be regarded as the source of heterogeneity and will be removed.

## Results

### Study selection and characteristics

**Fig. 1:**
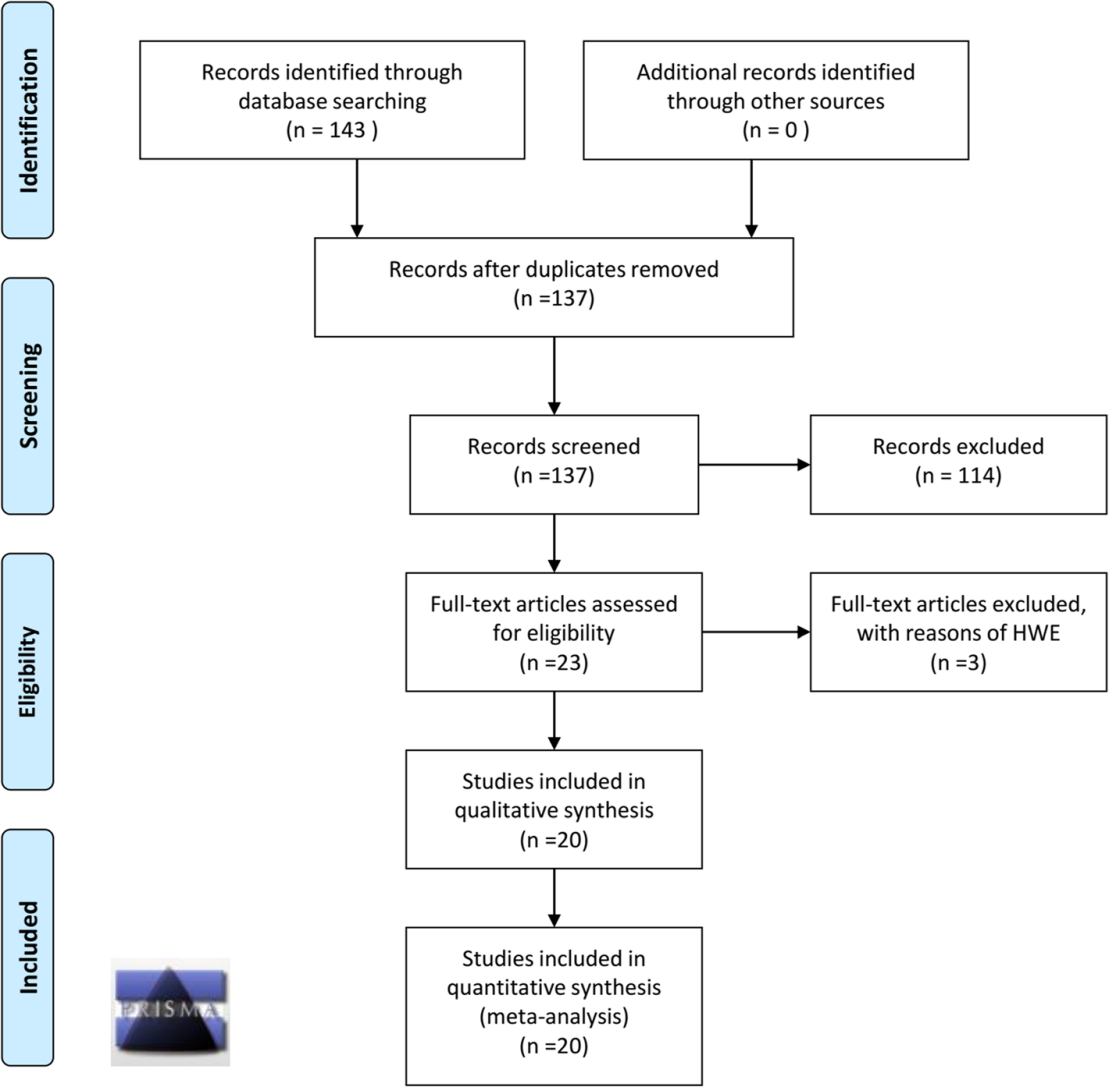
The PRISMA 2009 flow diagram of our study

Figure 1 shows the PRISMA 2009 flow diagram of our study. We obtained 143 records across three databases, including PUBMED (*n* = 31), WOS (*n* = 84) and EMBASE (*n* = 28). Then, a total of 137 records were screened after the duplicates were removed. After screening the titles and abstracts, 114 records were excluded for various reasons: animal or cell data, other disease or unconfirmed OSCC (n = 31); other genes, other variants or an unconfirmed *TP53* mutation site (*n* = 35); lack of a control group or full genotype frequency data in both the case and the control group (*n* = 21); meta-analysis, review, and meeting abstracts (*n* = 27). Next, the eligibility of 23 full-text articles was evaluated. From these articles, the genotype distributions of three articles did not adhere to HWE. Finally, a total of twenty articles were rigorously included in our quantitative synthesis. Of these twenty articles, sixteen studies [11–15, 19–29] examined oral squamous cell carcinoma (OSCC), and five studies [10, 11, 30–32] examined oral leukoplakia (OL). The basic information and genotype frequency of the included studies are listed in Additional file 3 and Additional file 4. NOS assessment system data (Additional file 5) showed that all of the enrolled case-control studies are high quality because all NOS quality scores were larger than five.

### TP53 rs1042522 and OSCC risk

**Fig. 2:**
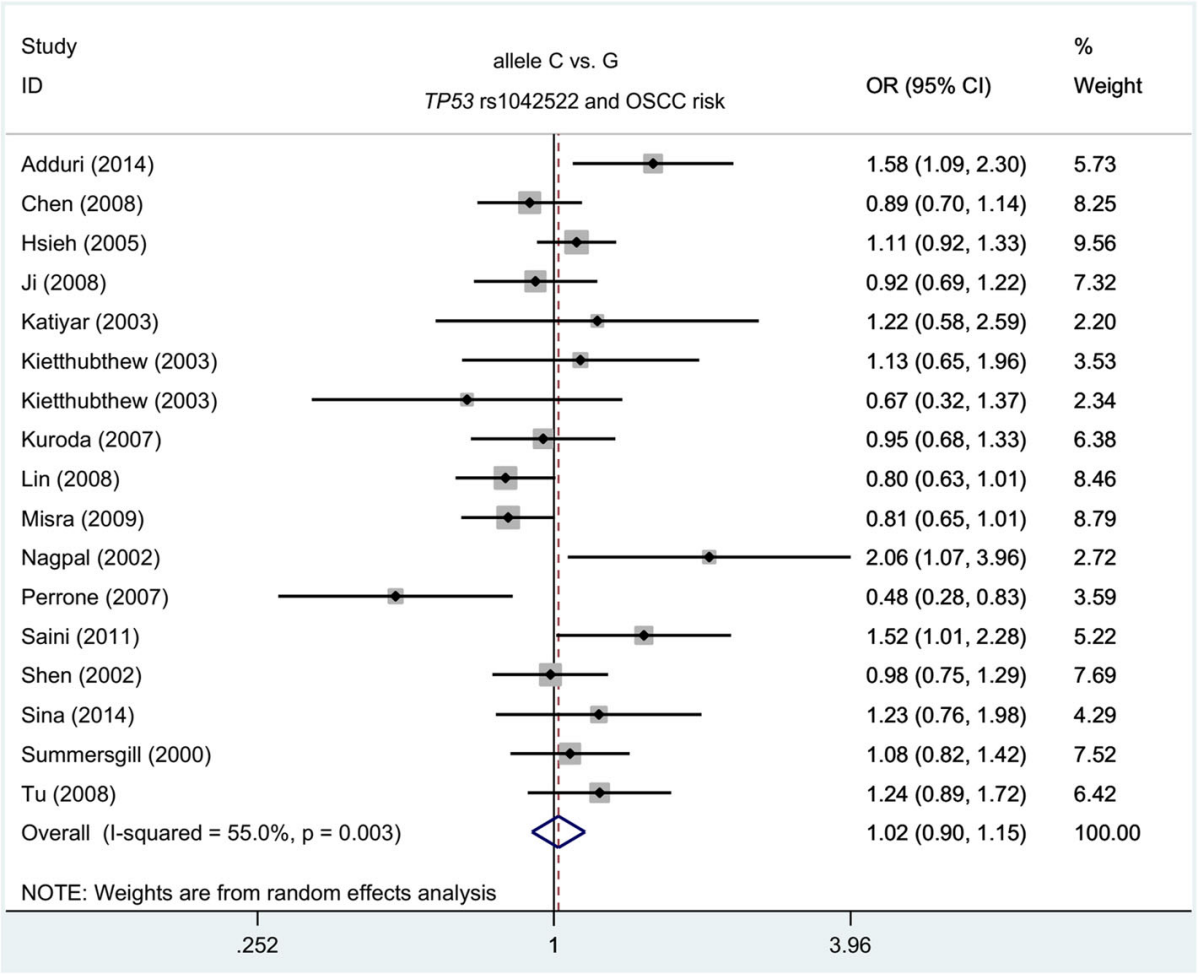
Meta-analysis (allele C vs. G) of *TP53* rs1042522 and OSCC risk

First, a total of 17 case-control studies from 16 articles with 3047 cases and 3305 controls were recruited for the meta-analysis of *TP53* rs1042522 and OSCC risk. Table 1 shows the heterogeneity for the three genetic models: allele C vs. G [I^2^ = 55.0%, *P*_H_ (*P-*value of heterogeneity = 0.003) and dominant GC + CC vs. GG **(**I^2^ = 47.6%, *P*_H_ = 0.015**)**, which led to the use of a random-effects model (DerSimonian and Laird method). A fixed-effects model (Mantel-Haenszel method) was utilized for others. The summary data in Table 1 show that compared with the controls, no increased or decreased risk of OSCC was observed in the cases for the six genetic models including allele C vs. G [*P*_*A*_ (*P*-value of association test) =0.741], carrier C vs. G (*P*_*A*_ = 0.853), homozygote CC vs. GG (*P*_*A*_ = 0.085), heterozygote GC vs. GG (*P*_*A*_ = 0.882), dominant GC + CC vs. GG (*P*_*A*_ = 0.969), and recessive CC vs. GG + GC (*P*_*A*_ = 0.980). Forest plot data of the allele C vs. G model are depicted in Fig. 2.

**Table 1:** Meta-analysis of *TP53* rs1042522 and OSCC risk

Genetic models	Overall/Subgroup		N	Case/control	ORs (95% CIs)	*P*_A_	I^2^ (%)	*P*_H_	Statistical model
allele C vs. G	overall		17	3047/3305	1.02 (0.90, 1.15)	0.741	55.0	0.003	Random
	Control source	PB	13	2477/2485	1.01 (0.86, 1.18)	0.925	63.7	0.001	
		HB	3	381/704	1.01 (0.83, 1.22)	0.933	0.0	0.663	
	Location	India	4	577/498	1.29 (0.79, 2.08)	0.306	79.1	0.002	
		USA	4	942/1357	0.96 (0.84, 1.10)	0.560	0.0	0.768	
		China	3	1115/767	1.02 (0.79, 1.31)	0.886	68.5	0.042	
	Ethnicity	Asian	12	2028/1807	1.10 (0.93, 1.29)	0.253	58.4	0.006	
		Caucasian	4	817/1165	0.86 (0.70, 1.06)	0.158	46.0	0.135	
	Disease type	oral cavity	6	1295/1372	1.12 (0.92, 1.36)	0.244	59.5	0.030	
		HPV16(−)	4	208/255	1.06 (0.47, 2.40)	0.884	83.4	< 0.001	
		HPV16(+)	4	93/197	1.38 (0.88, 2.16)	0.157	0.0	0.865	
carrier C vs. G	overall		17	3047/3305	0.99 (0.91, 1.08)	0.853	0.0	0.460	Fixed
	Control source	PB	13	2477/2485	0.98 (0.89, 1.08)	0.685	20.0	0.242	
		HB	3	381/704	1.00 (0.80, 1.25)	0.990	0.0	0.878	
	Location	India	4	577/498	1.02 (0.84, 1.25)	0.820	40.6	0.168	
		USA	4	942/1357	0.97 (0.83, 1.12)	0.666	0.0	0.902	
		China	3	1115/767	1.01 (0.87, 1.18)	0.869	8.9	0.334	
	Ethnicity	Asian	12	2028/1807	1.03 (0.92, 1.15)	0.601	0.0	0.600	
		Caucasian	4	817/1165	0.89 (0.76, 1.05)	0.179	35.6	0.199	
	Disease type	oral cavity	6	1295/1372	1.06 (0.93, 1.20)	0.414	0.0	0.510	
		HPV16(−)	4	208/255	0.94 (0.66, 1.34)	0.729	69.4	0.020	
		HPV16(+)	4	93/197	1.20 (0.71, 2.01)	0.493	0.0	0.926	
homozygote CC vs. GG	overall		17	3047/3305	1.03 (0.88, 1.21)	0.085	33.9	0.733	Fixed
	Control source	PB	13	2477/2485	1.00 (0.83, 1.20)	0.989	45.0	0.040	
		HB	3	381/704	1.06 (0.69, 1.62)	0.799	0.0	0.587	
	Location	India	4	577/498	1.04 (0.74, 1.47)	0.812	75.6	0.006	
		USA	4	942/1357	0.97 (0.70, 1.34)	0.846	0.0	0.945	
		China	3	1115/767	1.00 (0.76, 1.32)	0.997	65.3	0.056	
	Ethnicity	Asian	12	2028/1807	1.06 (0.88, 1.28)	0.559	53.1	0.015	
		Caucasian	4	817/1165	0.91 (0.63, 1.31)	0.602	0.0	0.972	
	Disease type	oral cavity	6	1295/1372	1.16 (0.92, 1.46)	0.224	56.1	0.044	
		HPV16(−)	4	208/255	1.46 (0.79, 2.73)	0.230	51.4	0.103	
		HPV16(+)	4	93/197	2.40 (0.96, 5.99)	0.061	0.0	0.843	
heterozygote GC vs. GG	overall		17	3047/3305	0.99 (0.89, 1.11)	0.882	37.4	0.061	Fixed
	Control source	PB	13	2477/2485	0.98 (0.86, 1.11)	0.706	50.1	0.020	
		HB	3	381/704	0.98 (0.75, 1.28)	0.861	0.0	0.969	
	Location	India	4	577/498	1.10 (0.82, 1.48)	0.541	0.0	0.402	
		USA	4	942/1357	0.94 (0.79, 1.12)	0.470	0.0	0.709	
		China	3	1115/767	1.10 (0.89, 1.35)	0.386	61.0	0.076	
	Ethnicity	Asian	12	2028/1807	1.10 (0.95, 1.28)	0.196	0.0	0.595	
		Caucasian	4	817/1165	0.81 (0.67, 0.98)	**0.030**	71.5	0.014	
	Disease type	oral cavity	6	1295/1372	1.15 (0.96, 1.38)	0.125	0.0	0.715	
		HPV16(−)	4	208/255	0.93 (0.30, 2.92)	0.904	80.6	0.001	
		HPV16(+)	4	93/197	0.96 (0.32, 2.86)	0.937	40.9	0.166	
dominant GC + CC vs. GG	overall		17	3047/3305	1.01 (0.86, 1.19)	0.969	47.6	0.015	Random
	Control source	PB	13	2477/2485	1.00 (0.81, 1.23)	0.936	57.5	0.005	
		HB	3	381/704	1.01 (0.86, 1.19)	0.181	0.0	0.877	
	Location	India	4	577/498	1.31 (0.77, 2.21)	0.321	57.8	0.068	
		USA	4	942/1357	0.94 (0.80, 1.11)	0.481	0.0	0.700	
		China	3	1115/767	1.07 (0.72, 1.58)	0.736	70.5	0.034	
	Ethnicity	Asian	12	2028/1807	1.13 (0.93, 1.36)	0.220	32.9	0.127	
		Caucasian	4	817/1165	0.78 (0.56, 1.07)	0.123	64.2	0.039	
	Disease type	oral cavity	6	1295/1372	1.15 (0.95, 1.40)	0.144	16.2	0.309	
		HPV16(−)	4	208/255	1.04 (0.32, 3.37)	0.942	83.9	< 0.001	
		HPV16(+)	4	93/197	1.21 (0.62, 2.37)	0.573	0.0	0.513	
recessive CC vs. GG + GC	overall		17	3047/3305	1.00 (0.87, 1.16)	0.980	22.8	0.189	Fixed
	Control source	PB	13	2477/2485	0.98 (0.84, 1.15)	0.794	36.4	0.092	
		HB	3	381/704	1.06 (0.71, 1.59)	0.765	0.0	0.560	
	Location	India	4	577/498	1.00 (0.75, 1.33)	0.997	75.6	0.006	
		USA	4	942/1357	0.99 (0.72, 1.36)	0.954	0.0	0.978	
		China	3	1115/767	0.95 (0.74, 1.21)	0.652	13.5	0.315	
	Ethnicity	Asian	12	2028/1807	1.00 (0.85, 1.18)	0.977	46.3	0.039	
		Caucasian	4	817/1165	0.98 (0.69, 1.40)	0.927	0.0	0.977	
	Disease type	oral cavity	6	1295/1372	1.00 (0.85, 1.18)	0.392	57.6	0.038	
		HPV16(−)	4	208/255	1.00 (0.85, 1.18)	0.353	16.3	0.310	
		HPV16(+)	4	93/197	1.00 (0.85, 1.18)	**0.031**	0.0	0.445	

*OSCC*, oral squamous cell carcinoma; *HPV*, Human papillomavirus; *N*, number of case-control studies; *PB*, population-based control; *HB*, hospital-based control; *HPV*, Human papillomavirus; *ORs*, odd ratios; *CIs*, confidence intervals; *P*_A,_ *P* value of association test; *P*_H,_ *P* values of heterogeneity test

*P*_A_ value <0.05, the number is in bold

Subgroup meta-analyses were also performed by PB (population-based)/HB (hospital-based), Caucasian/Asian, India/USA/China, and oral cavity/HPV16 (−)/HPV16(+). As shown in Table 1 and Additional file 6, Additional file 7, Additional file 8, Additional file 9, there is no statistically significant difference between the cases and the controls in all of the subgroup analyses (all *P*_*A*_ > 0.05) except for the Caucasian subgroup for the heterozygote model (*P*_A_=0.030) and the HPV16(+) subgroup for the recessive model (*P*_*A*_ = 0.031). These results indicate that *TP53* rs1042522 may have no significant influence on the risk of oral squamous cell carcinoma.

### TP53 rs1042522 and OL risk

**Fig. 3:**
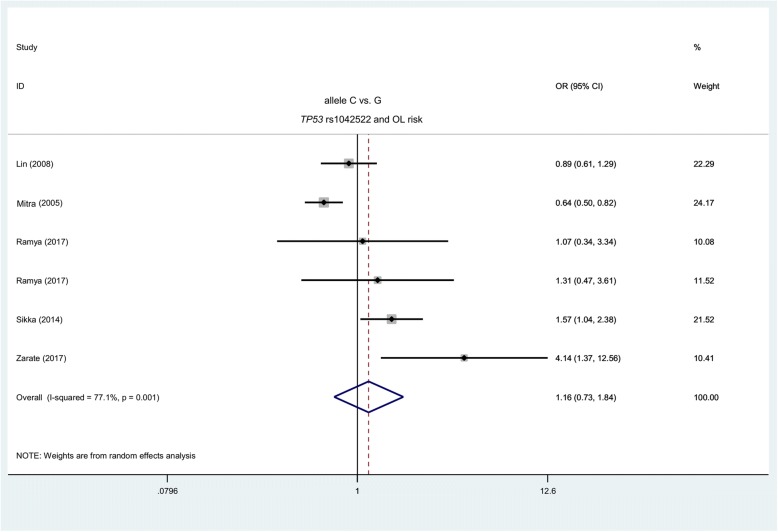
Meta-analysis (allele C vs. G) of *TP53* rs1042522 and OL risk

Six case-control studies with 391 cases and 763 controls were included from five articles for the meta-analysis of *TP53* rs1042522 and OL risk. As shown in Table 2, a fixed-effects model (Mantel-Haenszel method) was used for the carrier (I^2^ = 41.0%, *P*_H_ = 0.132) and heterozygote (I^2^ = 45.2%, *P*_H_ = 0.104) models, whereas a random-effects model (DerSimonian and Laird method) was used for the other alleles (I^2^ > 50.0%). We did not detect a difference between the cases and the controls for all of the genetic models in the overall meta-analysis (Table 2, *P*_*A*_ > 0.05). After stratification by PB, India and Asia, similar negative results were detected (Table 2, *P*_*A*_ > 0.05) and only separate from the homozygote (*P*_*A*_ = 0.003) and recessive (*P*_*A*_ = 0.004) model of PB subgroup. The forest plots are illustrated in Fig. 3 and in Additional file 10, Additional file 11, Additional file 12. These findings suggest that *TP53* rs1042522 may not be associated with the susceptibility to oral leukoplakia.

**Table 2:** Meta-analysis of *TP53* rs1042522 and OL risk

Genetic models	Overall/Subgroup		N	Case/control	ORs (95% CIs)	*P*_A_	I^2^ (%)	*P*_H_	Statistical model
allele C vs. G	Overall		6	391/763	1.16 (0.73, 1.84)	0.525	77.1	0.001	Random
	Control source	PB	4	291/647	0.77 (0.59, 1.01)	0.055	21.7	0.280	
	Location	India	4	307/465	1.05 (0.57, 1.92)	0.879	78.7	0.003	
	Ethnicity	Asian	5	377/745	0.99 (0.65, 1.49)	0.952	71.8	0.007	
carrier C vs. G	overall		6	391/763	0.93 (0.76, 1.15)	0.510	41.0	0.132	Fixed
	Control source	PB	4	291/647	0.82 (0.65, 1.03)	0.090	0.0	0.713	
	Location	India	4	307/465	0.89 (0.70, 1.13)	0.353	39.1	0.177	
	Ethnicity	Asian	5	377/745	0.90 (0.73, 1.11)	0.334	19.3	0.292	
homozygote CC vs. GG	overall		6	391/763	1.14 (0.49, 2.62)	0.764	69.2	0.006	Random
	Control source	PB	4	291/647	0.52 (0.34, 0.80)	**0.003**	1.0	0.387	
	Location	India	4	307/465	1.06 (0.33, 3.37)	0.920	75.6	0.006	
	Ethnicity	Asian	5	377/745	0.93 (0.41, 2.08)	0.854	67.5	0.015	
heterozygote GC vs. GG	overall		6	391/763	0.95 (0.71, 1.29)	0.760	45.2	0.104	Fixed
	Control source	PB	4	291/647	0.85 (0.61, 1.18)	0.330	0.0	0.700	
	Location	India	4	307/465	0.81 (0.57, 1.15)	0.239	0.0	0.827	
	Ethnicity	Asian	5	377/745	0.87 (0.64, 1.19)	0.388	0.0	0.803	
dominant GC + CC vs. GG	overall		6	391/763	1.20 (0.66, 2.18)	0.557	63.1	0.019	Random
	Control source	PB	4	291/647	0.73 (0.53, 1.00)	0.050	0.0	0.460	
	Location	India	4	307/465	0.88 (0.51, 1.50)	0.629	32.0	0.220	
	Ethnicity	Asian	5	377/745	0.87 (0.60, 1.25)	0.446	21.0	0.281	
recessive CC vs. GG + GC	overall		6	391/763	1.04 (0.53, 2.05)	0.907	68.9	0.007	Random
	Control source	PB	4	291/647	0.59 (0.41, 0.85)	**0.004**	0.0	0.505	
	Location	India	4	307/465	1.11 (0.42, 2.98)	0.829	79.7	0.002	
	Ethnicity	Asian	5	377/745	0.97 (0.47, 2.01)	0.943	73.6	0.004	

OL, oral leukoplakia; N, number of case-control studies; PB: population-based control; ORs, odd ratios; CIs, confidence intervals; *P*_A,_ *P* value of association test; *P*_H,_ *P* values of heterogeneity test

*P*_A_ value <0.05, the number is in bold

### Publication bias and sensitivity analysis

**Fig. 4:**
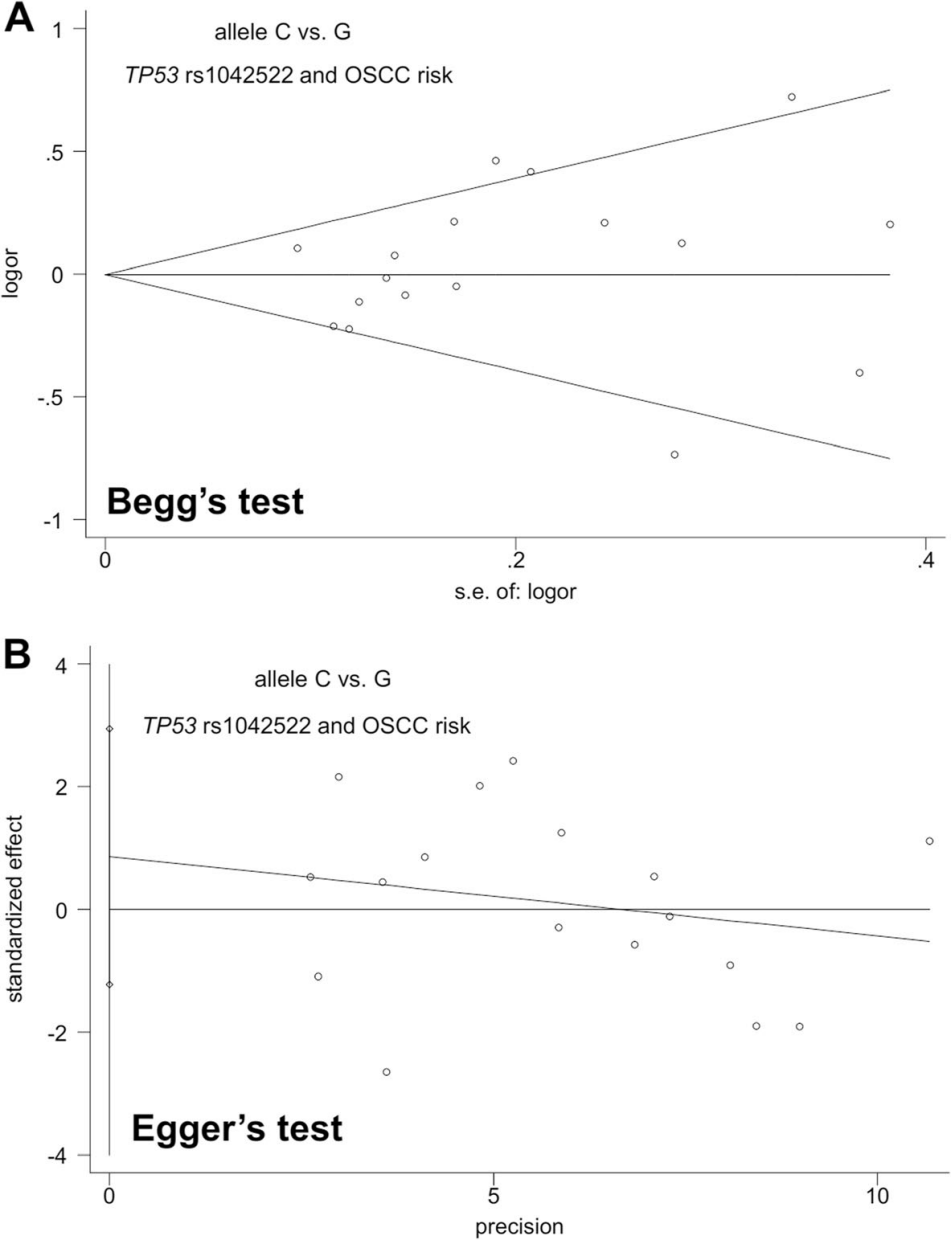
Publication bias evaluation (allele C vs. G) of *TP53* rs1042522 and OSCC risk. (**a**) Begg’s test; (**b**) Egger’s test

We performed both the Begg’s test and Egger’s test to qualitatively assess the presence of publication bias. Because no more than ten case-control studies in this meta-analysis examined OL, we only analyzed the publication bias in the meta-analysis of OSCC. As shown in Table 3, the *P-*value of Begg’s test and Egger’s test was larger than 0.05 for all the above genetic models [*P*_*B*_ (*P*-value of Begg’s test) > 0.05; *P*_*E*_ (*P*-value of Egger’s test) > 0.05]. The Begg’s funnel plot (Fig. 4A) and Egger’s publication bias plot (Fig. 4B) of the allele model are shown as an example. Thus, there was no large publication bias in our study.

**Table 3:** Publication bias evaluation

Genetic models	Begg’s test*		Egger’s test	
	z	*P*_*B*_	t	*P*_*E*_
allele C vs. G	1.03	0.303	0.88	0.393
carrier C vs. G	0.78	0.434	0.52	0.609
homozygote CC vs. GG	0.78	0.434	1.51	0.152
heterozygote GC vs. GG	0.95	0.343	0.19	0.856
dominant GC + CC vs. GG	0.78	0.434	0.69	0.504
recessive CC vs. GG + GC	0.87	0.387	1.63	0.124

^*^continuity corrected; *OSCC*, oral squamous cell carcinoma;

*OL*, oral leukoplakia; *P*_*B*_*, P* value of Begg’s test; *P*_*E*_*, P* value of Egger’s test

**Fig. 5:**
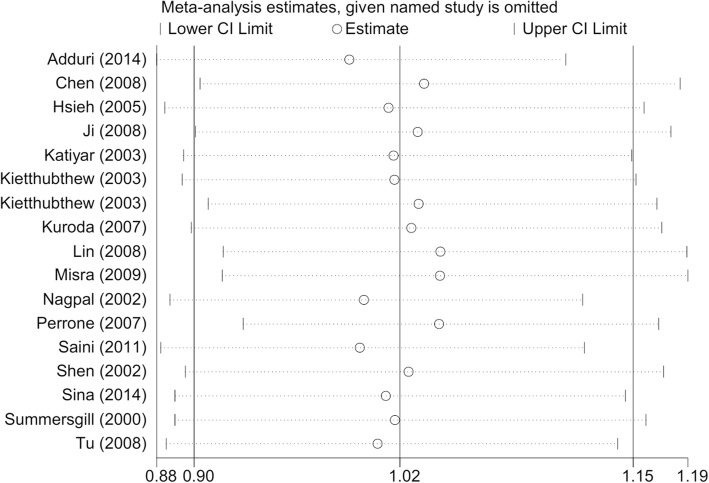
Sensitivity analysis (allele C vs. G) of *TP53* rs1042522 and OSCC risk

Moreover, we observed a similar summarized OR value in our sensitivity analysis (Fig. 5 for the allele model of OSCC; Additional file 13 for the allele model of OL; and other data not shown), which indicated the reliability of our results.

## Discussion

In this study, we focused on the potential role of *TP53* rs1042522 in the risk of oral squamous cell carcinoma through a meta-analysis of sixteen case-control studies. Overall, the results of the present meta-analysis failed to find any significant association (*P*-value of the association test> 0.05) between *TP53* rs1042522 and the risk of OSCC in either the Asian or Caucasian population. Additionally, the current meta-analysis investigated the potential role of *TP53* rs1042522 in oral leukoplakia risk based on all the published articles that were available. The results showed that *TP53* rs1042522 may not be a susceptible factor for oral leukoplakia disease.

Our meta-analysis data of OSCC coincides with the results reported earlier [16, 17]. In 2009, Zhou et al. performed the first meta-analysis of nine studies [11, 13, 14, 19–21, 23, 25, 28] and found that *TP53* rs1042522 does not seem to be associated with the risk of OSCC [16]. In 2014, Zeng et al. selected eleven case-control studies [11, 13, 19, 22, 23, 26, 28, 29, 33–35] for a meta-analysis regarding the role of *TP53* rs1042522 in the risk of OSCC risk among the Asian population and reported that *TP53* rs1042522 is not linked to the risk of an HPV-negative OSCC patient among Asians [17]. In the present meta-analysis, we worked toward identifying the effect *TP53* rs1042522 on the risk of OSCC in not only the Asian population but also the Caucasian population. We removed one study [33], in which oral cancer was not histopathologically confirmed as SCC, and two other studies [34, 35] for deviation from the Hardy-Weinberg equilibrium. More importantly, we added another eight new case-control studies [12, 14, 15, 20, 21, 24, 25, 27] in our updated meta-analysis.

Despite the above negative association between *TP53* rs1042522 and OSCC risk, different conclusions were observed in meta-analyses regarding the genetic relationship between *TP53* rs1042522 and oral cancer risk [36, 37]. In 2013, Jiang et al. identified 17 case-control studies [11, 13, 14, 19–23, 25, 26, 28, 29, 33, 35, 38–40] for a meta-analysis and reported a lack of a genetic link between *TP53* rs1042522 and oral cancer risk [36]. However, In 2015, Hou et al. statistically pooled 13 studies [11, 19, 20, 22–24, 26, 39, 41–45] for another meta-analysis of the association between *TP53* rs1042522 and oral cancer and revealed that *TP53* rs1042522 may be linked to the pathogenesis of oral cancer [37]. Among these included studies, we noted that several case-control studies [33, 39, 40] do not provide the pathological typing information of oral cancer; however, OSCC accounts for most of oral cancer cases. In addition, the genotype distributions of the control group in two studies [35, 38] were not in line with Hardy-Weinberg Equilibrium.

Our updated meta-analysis enrolled as much articles as possible. Strict inclusion and exclusion criteria were utilized to select the eligible case-control studies. The reliability of our results was also observed in our sensitivity analysis. However, the limitations still exist in our study. The following concerns should be addressed. (1) Our statistical conclusion should be further verified by more case-control studies with a larger number of subjects. Only six case-control studies from five articles [10, 11, 30–32] were included for the meta-analysis of oral leukoplakia, and only four case-control studies [13–15, 26] were enrolled in the HPV 16 +/− subgroup meta-analysis of OSCC. We only detected the role of HPV 16 but not any other type of HPV. In addition, we only enrolled four case-control studies [15, 20, 21, 24] for the “non-Asian, Caucasian” subgroup analysis of *TP53* rs1042522 and OSCC risk. Furthermore, no case-control study population was obtained for the “Caucasian” subgroup analysis of *TP53* rs1042522 and OL risk. (2) The existence of between-study heterogeneity was observed in some comparisons. For example, the high heterogeneity among the case-control studies in the overall meta-analysis of *TP53* rs1042522 and OSCC risk under allele and dominant genetic models disappears in the hospital-based, USA and HPV16(+) subgroups. The complexity of OSCC/OL pathogenesis, the source of control, location and ethnicity may be involved in this dynamic. (3) We did not perform the meta-analysis regarding the role of the other loci of the *TP53* gene or the variant combination between the *TP53* gene and other genes. (4) No case-control study in the Caucasian population was enrolled in the meta-analysis of *TP53* rs1042522 and OL risk. In addition, we did not perform Begg’s test and Egger’s test to assess the risk of publication bias in meta-analysis of OL because the number of included case-control studies was less than ten. Even though our data from Begg’s test and Egger’s test show no proof of publication bias for the meta-analysis of OSCC, we still cannot ignore the impact of publication language, time, and regional variation on the presence of selection bias. (5) Even though the basic information of gender, age, smoking and alcohol consumption was gathered, the relevant stratification analyses by adjusted factors were not performed due to the lack of original genotype frequency data in both the case and control groups.

## Conclusions

In conclusion, according to the currently available case-control studies, our updated meta-analysis data together with previous reports fail to statistically support the genetic relationship between *TP53* rs1042522 and the risk of oral squamous cell carcinoma. Additionally, our meta-analysis is the first study to report that the *TP53* rs1042522 polymorphism does not appear to confer susceptibility to oral leukoplakia patients. Additional high-quality case-control studies will help us to scientifically assess the significance of the *TP53* rs1042522 polymorphism on the risk of oral leukoplakia and oral squamous cell carcinoma.

## Additional files

Additional file 1: PRISMA 2009 checklist. (DOCX 28 kb)

Additional file 2: The terms of the database search. (DOCX 18 kb)

Additional file 3: Basic information of the included case-control studies. (DOCX 21 kb)

Additional file 4: Genotype frequency data of the included case-control studies. (DOCX 19 kb)

Additional file 5: Quality assessment of the included case-control studies. (DOCX 23 kb)

Additional file 6: Subgroup analysis (allele C vs. G) of OSCC by the control source. (TIF 1495 kb)

Additional file 7: Subgroup analysis (allele C vs. G) of OSCC by the location. (TIF 1307 kb)

Additional file 8: Subgroup analysis (allele C vs. G) of OSCC by the ethnicity. (TIF 1362 kb)

Additional file 9: Subgroup analysis (allele C vs. G) of OSCC by the disease type. (TIF 1498 kb)

Additional file 10: Subgroup analysis (allele C vs. G) of OL by the location. (TIF 1225 kb)

Additional file 11: Subgroup analysis (allele C vs. G) of OL by the ethnicity. (TIF 1092 kb)

Additional file 12: Subgroup analysis (allele C vs. G) of OL by the control source. (TIF 1103 kb)

Additional file 13: Sensitivity analysis (allele C vs. G) of *TP53* rs1042522 and OL risk. (TIF 358 kb)

## Abbreviations

CI: confidence interval
Embase: Excerpta Medica Database
HPV: papillomavirus
HWE: Hardy-Weinberg Equilibrium
OL: oral leukoplakia
OPMD: oral potentially malignant disorders
ORs: odd ratios
OSCC: oral squamous cell carcinoma
PRISMA: preferred reporting items for systematic reviews and meta-analyses
TP53: tumor suppressor p53
WOS: Web of Science
