# Supplementary material for: Effect of TP53 rs1042522 on the susceptibility of patients to oral squamous cell carcinoma and oral leukoplakia: a meta-analysis
Source: BMC Oral Health. 2018 Aug 20;18:143. doi: 10.1186/s12903-018-0603-6 (PMC6102817; doi:10.1186/s12903-018-0603-6)
Supplement: Supplementary file 2 — The terms of the database search. (DOCX 18 kb) [file 12903_2018_603_MOESM2_ESM.docx]

**Additional file 2** The terms of the database search

| **Database** | **#** | **Query** | **Record** |
| --- | --- | --- | --- |
| **PUBMED** | | | |
|  | **#1** | ((((((((Leukoplakia, Oral[MeSH Terms]) OR Leukoplakias, Oral) OR Oral Leukoplakia) OR Oral Leukoplakias) OR Leukokeratosis, Oral) OR Leukokeratoses, Oral) OR Oral Leukokeratoses) OR Oral Leukokeratosis) OR Keratosis, Oral | 5,635 |
|  | **#2** | (Oral squamous cell carcinoma) OR OSCC | 28,898 |
|  | **#3** | (#1 OR #2) | 33,028 |
|  | **#4** | (((((((((Genes, p53[MeSH Terms]) OR TP53 Genes) OR Genes, TP53) OR Gene, TP53) OR TP53 Gene) OR p53 Genes) OR Gene, p53) OR p53 Gene) OR TP53) OR P53 | 9,5066 |
|  | **#5** | ((((((Polymorphism, Single Nucleotide [MeSH Terms]) OR Nucleotide Polymorphism, Single) OR Nucleotide Polymorphisms, Single) OR Polymorphisms, Single Nucleotide) OR Single Nucleotide Polymorphisms) OR SNPs) OR Single Nucleotide Polymorphism | 12,6825 |
|  | **#6** | (#3 AND #4 AND #5)  (((((((((((((Leukoplakia, Oral[MeSH Terms]) OR Leukoplakias, Oral) OR Oral Leukoplakia) OR Oral Leukoplakias) OR Leukokeratosis, Oral) OR Leukokeratoses, Oral) OR Oral Leukokeratoses) OR Oral Leukokeratosis) OR Keratosis, Oral)) OR ((Oral squamous cell carcinoma) OR OSCC))) AND (((((((Polymorphism, Single Nucleotide [MeSH Terms]) OR Nucleotide Polymorphism, Single) OR Nucleotide Polymorphisms, Single) OR Polymorphisms, Single Nucleotide) OR Single Nucleotide Polymorphisms) OR SNPs) OR Single Nucleotide Polymorphism)) AND ((((((((((Genes, p53[MeSH Terms]) OR TP53 Genes) OR Genes, TP53) OR Gene, TP53) OR TP53 Gene) OR p53 Genes) OR Gene, p53) OR p53 Gene) OR TP53) OR P53) | 31 |
| **WOS** | | | |
|  | **#1** | TOPIC: (Leukoplakia, Oral) OR TOPIC: (Leukoplakias, Oral) OR TOPIC: (Oral Leukoplakia) OR TOPIC: (Oral Leukoplakias) OR TOPIC: (Leukokeratosis, Oral) OR TOPIC: (Leukokeratoses, Oral) OR TOPIC: (Oral Leukokeratoses) OR TOPIC: (Oral Leukokeratosis) OR TOPIC: (Keratosis, Oral) | 3,645 |
|  | **#2** | TOPIC: (Oral squamous cell carcinoma) OR TOPIC: (OSCC) | 28,908 |
|  | **#3** | (#1 OR #2) | 31,124 |
|  | **#4** | OPIC: (Genes, p53) OR TOPIC: (TP53 Genes) OR TOPIC: (Genes, TP53) OR TOPIC: (Gene, TP53) OR TOPIC: (TP53 Gene) OR TOPIC: (p53 Genes) OR TOPIC: (Gene, p53) OR TOPIC: (TP53) OR TOPIC: (P53) | 121,234 |
|  | **#5** | TOPIC: (Polymorphism, Single Nucleotide) OR TOPIC: (Nucleotide Polymorphism, Single) OR TOPIC: (Nucleotide Polymorphisms, Single) OR TOPIC: (Polymorphisms, Single Nucleotide) OR TOPIC: (Single Nucleotide Polymorphisms) OR TOPIC: (SNPs) OR TOPIC: (Single Nucleotide Polymorphism) | 197,231 |
|  | **#6** | (#3 AND #4 AND #5) | 84 |
| **EMBASE** | | | |
|  | **#1** | leukoplakia'/exp OR 'abdominal sepsis' OR 'leucokeratosis nicrotinica paltina' OR 'leucoplakia; leucoplakia, oral' OR 'leucoplakia, mouth' OR 'leukokeratosis' OR 'leukokeratosis nicrotinica paltina' OR 'leukoplakia, oral' OR 'leukoplakia, mouth' OR 'leukoplasia' OR 'mouth leucoplakia' OR 'mouth leukoplakia' OR 'mouth white lesion'/exp OR 'mouth white lesion' OR 'oral lesion, precancerous'/exp OR 'oral lesion, precancerous' OR 'oral lesion, premalignant'/exp OR 'oral lesion, premalignant' OR 'oral leucoplakia'/exp OR 'oral leucoplakia' OR 'oral precancerous condition'/exp OR 'oral precancerous condition' OR 'oral white lesion'/exp OR 'oral white lesion' OR 'precancerous oral lesion'/exp OR 'precancerous oral lesion' OR 'precarcinoma, mouth'/exp OR 'precarcinoma, mouth' OR 'premalignant oral lesion'/exp OR 'premalignant oral lesion' OR 'white lesion, mouth'/exp OR 'white lesion, mouth' | 9,150 |
|  | **#2** | 'mouth squamous cell carcinoma'/exp OR 'oral squamous cell carcinoma'/exp OR 'oral squamous cell carcinoma' OR 'oscc' | 12,326 |
|  | **#3** | (#1 OR #2) | 20,784 |
|  | **#4** | 'protein p53'/exp OR 'p53' OR 'p53 protein' OR 'phosphoprotein p53' OR 'protein p 53' OR 'protein tp53' OR 'tp53 protein' OR 'tumor suppressor protein p53' OR 'tumour suppressor protein' | 135,482 |
|  | **#5** | 'single nucleotide polymorphism'/exp OR 'polymorphism, single nucleotide'/exp OR 'polymorphism, single nucleotide' OR 'single nucleotide variant'/exp OR 'single nucleotide variant' OR 'single nucleotide variation'/exp OR 'single nucleotide variation' | 150,988 |
|  | **#6** | (#3 AND #4 AND #5) | 28 |
