# Supplementary material for: Effect of TP53 rs1042522 on the susceptibility of patients to oral squamous cell carcinoma and oral leukoplakia: a meta-analysis
Source: BMC Oral Health. 2018 Aug 20;18:143. doi: 10.1186/s12903-018-0603-6 (PMC6102817; doi:10.1186/s12903-018-0603-6)
Supplement: Supplementary file 3 — Basic information of the included case-control studies. (DOCX 21 kb) [file 12903_2018_603_MOESM3_ESM.docx]

Additional file 3 Basic information of the included case-control studies.

| **First author (Year)** | **Disease type** | **Case/control** | **Method** | **Mean Age**  **(case/control)** | **Male (%)**  **(case/control)** | **Smoking**  **(%)** | **Alcohol (%)** |
| --- | --- | --- | --- | --- | --- | --- | --- |
| **Adduri (2014)** | OSCC | 115/110 | Gene sequencing | 33.9 /-^@^ | 66.1/- | 72.0/- | 53.3/- |
| **Chen (2008)** | OSCC | 326/349 | PCR-RFLP | 9.5/10.6^@^ | 74.2/77.9 | 69.6/69.3 | 76.6/73.4 |
| **Hsieh (2005)** | OSCC | 629/371 | PCR-RFLP | 49.93/- | - | - | - |
| **Ji (2008)** | OSCC | 188/342 | PCR-RFLP | 9.0/10.8^@^ | 82.4/80.7 | 66.5/68.7 | 79.8/74.3 |
| **Katiyar (2003)** | OSCC | 44/20 | PCR | - | - | - | - |
| **Kietthubthew (2003)** | OSCC | 82/81 | PCR-RFLP | 67.4/67.8 | 69.1/69.1 | 53.6/52.6 | 53.6/52.6 |
| **Kuroda (2007)** | OSCC | 100/271 | PCR-RFLP | 62.5/63.3 | 64.0/64.6 | 66.0/59.0 | - |
| **Lin (2008)** | OSCC | 297/280 | PCR-RFLP | 49.5/52.1 | 92.6/47.5 | 81.5/15.7 | 68.4/21.1 |
|  | OL | 70/280 | PCR-RFLP | 49.8/52.1 | 92.9/47.5 | 81.4/15.7 | 82.9/21.1 |
| **Misra (2009)** | OSCC | 308/342 | PCR-RFLP | 55.0/50.4 | 63.5/76.1 | 43.2/53.4 | 18.4/46.5 |
| **Mitra (2005)** | OL | 191/342 | PCR-RFLP | 47.0/50.4 | 86.3/76.1 | 96.5/53.4 | 15.7/46.5 |
| **Nagpal (2002)** | OSCC | 110/26 | PCR | 60.0/-^@@^ | 61.8/- | - | - |
| **Perrone (2007)** | OSCC | 77/141 | Double gradient-denaturing gel electrophoresis | 58.0/- | 76.6/56.0 | - | - |
| **Ramya (2017)** | OL* | 15/10 | PCR-RFLP | 46.7/40.0 | 80.0/60.0 | - | - |
|  | OL^&^ | 15/15 | PCR-RFLP | 46.7/50.5 | 80.0/80.0 | 60.0/66.7 | - |
| **Saini (2011)** | OSCC | 99/90 | PCR | 56.2/42.0 | 48.6/55.2 | 37.1/20.0 | 20.0/11.4 |
| **Shen (2002)** | OSCC | 226/333 | PCR-RFLP | 15.1/13.2^@@@^ | 75.0/68.8 | 74.7/69.6 | 79.6/69.4 |
| **Sikka (2014)** | OL | 86/98 | PCR-RFLP | 48.0/49.0 | 90.1/87.0 | 45.0/59.3 | - |
| **Sina (2014)** | OSCC-oral cavity | 55/100 | PCR-ARMS | 65.9/62.7 | 52.7/50.0 | - | - |
| **Summersgill (2000)** | OSCC | 202/333 | PCR | 5.0/9.9^@^ | 65.4/58.9 | - | - |
| **Tu (2008)** | OSCC | 189/116 | Gene sequencing | 51.4/46.4 | 100/100 | - | - |
| **Zarate (2017)** | OL | 14/18 | PCR-RFLP | 9.1/11.1^@@@^ | 63.6/50.0 | - | - |

OSCC, oral squamous cell carcinoma; OL, oral leukoplakia; *, healthy individuals without any deleterious oral habits; &, healthy individuals with deleterious oral habits; PCR: polymerase chain reaction; PCR-RFLP: polymerase chain reaction-restriction fragment length polymorphism; PCR-SSP: polymerase chain reaction-sequence specific primer; PCR- ARMS, polymerase chain reaction-amplification refractory mutation system; ^@^, age less than 40 years (%); ^@@^, age less than 50 years (%); ^@@@^, age less than 45 years (%);-, not available.
