# Supplementary material for: Effect of TP53 rs1042522 on the susceptibility of patients to oral squamous cell carcinoma and oral leukoplakia: a meta-analysis
Source: BMC Oral Health. 2018 Aug 20;18:143. doi: 10.1186/s12903-018-0603-6 (PMC6102817; doi:10.1186/s12903-018-0603-6)
Supplement: Supplementary file 4 — Genotype frequency data of the included case-control studies. (DOCX 19 kb) [file 12903_2018_603_MOESM4_ESM.docx]

Additional file 4 Genotype frequency data of the included case-control studies.

| **First author (Year)** | **Case** | **Disease type** | **Control** | **Control source** | **Location** | **Ethnicity** |
| --- | --- | --- | --- | --- | --- | --- |
|  | **GG/GC/CC** |  | **GG/GC/CC** |  |  |  |
| **Adduri (2014)** | 23/48/44 | OSCC-oral cavity | 31/53/26 | PB | India | Asian |
| **Chen (2008)** | 183/121/22 | OSCC | 181/144/24 | PB | USA | Caucasian |
| **Hsieh (2005)** | 149/274/100 | OSCC-oral cavity | 128/177/66 | PB | China | Asian |
|  | 187/328/114 | OSCC-all | 128/177/66 | PB | China | Asian |
| **Ji (2008)** | 103/74/11 | OSCC-oropharynx | 179/140/23 | PB | USA | Caucasian |
| **Katiyar (2003)** | 2/10/1 | OSCC-HPV16(+) | 5/12/3 | PB | India | Asian |
|  | 8/14/9 | OSCC-HPV16(-) | 5/12/3 | PB | India | Asian |
| **Kietthubthew (2003)** | 16/25/11 | OSCC-smoke/drink(+) | 20/19/12 | PB | Thailand | Asian |
|  | 11/13/6 | OSCC-smoke/drink(-) | 9/11/10 | PB | Thailand | Asian |
| **Kuroda (2007)** | 41/44/15 | OSCC | 109/117/45 | HB | Japan | Asian |
| **Lin (2008)** | 96/155/46 | OSCC | 72/152/56 | PB | China | Asian |
|  | 18/42/10 | OL | 72/152/56 | PB | China | Asian |
| **Misra (2009)** | 87/155/66 | OSCC-oral cavity | 85/159/98 | PB | India | Asian |
| **Mitra (2005)** | 67/92/32 | OL | 85/159/98^*^ | PB | India | Asian |
| **Nagpal (2002)** | 17/38/14 | OSCC-HPV16(-) | 13/11/2 | PB | India | Asian |
|  | 8/13/4 | OSCC-HPV16(+) | 13/11/2 | PB | India | Asian |
| **Perrone (2007)** | 52/7/2 | OSCC-HPV16(-) | 84/47/10 | PB | Italy | Caucasian |
|  | 11/1/4 | OSCC-HPV16(+) | 84/47/10 | PB | Italy | Caucasian |
| **Ramya (2017)** | 3/7/5 | OL^&^ | 2/5/3 | PB | India | Asian |
|  | 3/7/5 | OL* | 4/7/4 | PB | India | Asian |
| **Saini (2011)** | 11/22/14 | OSCC-HPV16(-) | 23/28/17 | PB | Malaysia | Asian |
|  | 8/16/15 | OSCC-HPV16(+) | 2/6/2 | PB | Malaysia | Asian |
| **Shen (2002)** | 55/41/9 | OSCC-oral cavity | 175/134/24 | HB | USA | Caucasian |
|  | 121/88/17 | OSCC-all | 175/134/24 | HB | USA | Caucasian |
| **Sikka (2014)** | 14/38/34 | OL | 21/55/22 | HB | India | Asian |
| **Sina (2014)** | 20/25/10 | OSCC-oral cavity | 40/48/12 | HB | Iran | Asian |
| **Summersgill (2000)** | 31/21/6 | OSCC-HPV(+) | 36/20/6 | PB | USA | Mixed |
|  | 76/55/13 | OSCC-HPV(-) | 149/98/24 | PB | USA | Mixed |
| **Tu (2008)** | 53/106/30 | OSCC-oral cavity | 41/60/15 | NR | China | Asian |
| **Zarate (2017)** | 3/8/3 | OL | 13/3/2 | HB | Argentina | mixed |

OSCC, oral squamous cell carcinoma; OL, oral leukoplakia; HPV, human papillomavirus; PB: population-based control; HB: hospital-based control; NR, not reported; *, healthy individuals without any deleterious oral habits; &, healthy individuals with deleterious oral habits.
