# Supplementary material for: Effect of TP53 rs1042522 on the susceptibility of patients to oral squamous cell carcinoma and oral leukoplakia: a meta-analysis
Source: BMC Oral Health. 2018 Aug 20;18:143. doi: 10.1186/s12903-018-0603-6 (PMC6102817; doi:10.1186/s12903-018-0603-6)
Supplement: Supplementary file 5 — Quality assessment of the included case-control studies. (DOCX 23 kb) [file 12903_2018_603_MOESM5_ESM.docx]

**Additional file 5** Quality assessment of the included case-control studies.

| **First author (Year)** | **(1)** | **(2)** | **(3)** | **(4)** | **(5)** | **(6)** | **(7)** | **(8)** | **(9)** | **Score** |
| --- | --- | --- | --- | --- | --- | --- | --- | --- | --- | --- |
| **Adduri (2014)** | *1* | *1* | *1* | *1* | *1* | *1* | *1* | *1* | *1* | **9** |
| **Chen (2008)** | *0* | *1* | *1* | *1* | *1* | *1* | *1* | *1* | *1* | **8** |
| **Hsieh (2005)** | *1* | *1* | *1* | *1* | *0* | *1* | *1* | *1* | *1* | **8** |
| **Ji (2008)** | *1* | *1* | *1* | *1* | *1* | *1* | *1* | *1* | *1* | **9** |
| **Katiyar (2003)** | *0* | *1* | *1* | *1* | *1* | *0* | *1* | *1* | *1* | **7** |
| **Kietthubthew (2003)** | *1* | *1* | *1* | *1* | *1* | *1* | *1* | *1* | *0* | **8** |
| **Kuroda (2007)** | *0* | *1* | *0* | *0* | *1* | *1* | *1* | *1* | *1* | **6** |
| **Lin (2008)** | *1* | *1* | *1* | *1* | *1* | *1* | *1* | *1* | *1* | **9** |
| **Misra (2009)** | *1* | *1* | *1* | *1* | *0* | *1* | *1* | *1* | *0* | **7** |
| **Mitra (2005)** | *1* | *1* | *1* | *1* | *0* | *1* | *1* | *1* | *0* | **7** |
| **Nagpal (2002)** | *1* | *1* | *1* | *1* | *0* | *1* | *1* | *1* | *1* | **8** |
| **Perrone (2007)** | *0* | *1* | *1* | *1* | *0* | *1* | *0* | *1* | *1* | **6** |
| **Ramya (2017)** | *1* | *1* | *1* | *0* | *0* | *1* | *1* | *1* | *1* | **7** |
| **Saini (2011)** | *0* | *1* | *1* | *1* | *0* | *1* | *1* | *1* | *0* | **6** |
| **Shen (2002)** | *1* | *1* | *0* | *0* | *1* | *1* | *1* | *1* | *0* | **6** |
| **Sikka (2014)** | *1* | *1* | *0* | *0* | *1* | *1* | *1* | *1* | *0* | **6** |
| **Sina (2014)** | *1* | *1* | *0* | *0* | *1* | *1* | *0* | *1* | *1* | **6** |
| **Summersgill (2000)** | *0* | *1* | *1* | *1* | *1* | *1* | *0* | *1* | *1* | **7** |
| **Tu (2008)** | *1* | *1* | *0* | *0* | *1* | *1* | *1* | *1* | *1* | **7** |
| **Zarate (2017)** | *1* | *1* | *0* | *0* | *1* | *1* | *0* | *1* | *1* | **6** |

1. Case Definition; (2) Representativeness of cases; (3) Selection of controls; (4) Definition of controls; (5) Important factors of comparability; (6) Other factors of comparability; (7) Secure record of exposure; (8) Same method of ascertainment; (9) Non-response rate.
