# Supplementary figures and images for: Effect of TP53 rs1042522 on the susceptibility of patients to oral squamous cell carcinoma and oral leukoplakia: a meta-analysis
Source: BMC Oral Health. 2018 Aug 20;18:143. doi: 10.1186/s12903-018-0603-6 (PMC6102817; doi:10.1186/s12903-018-0603-6)

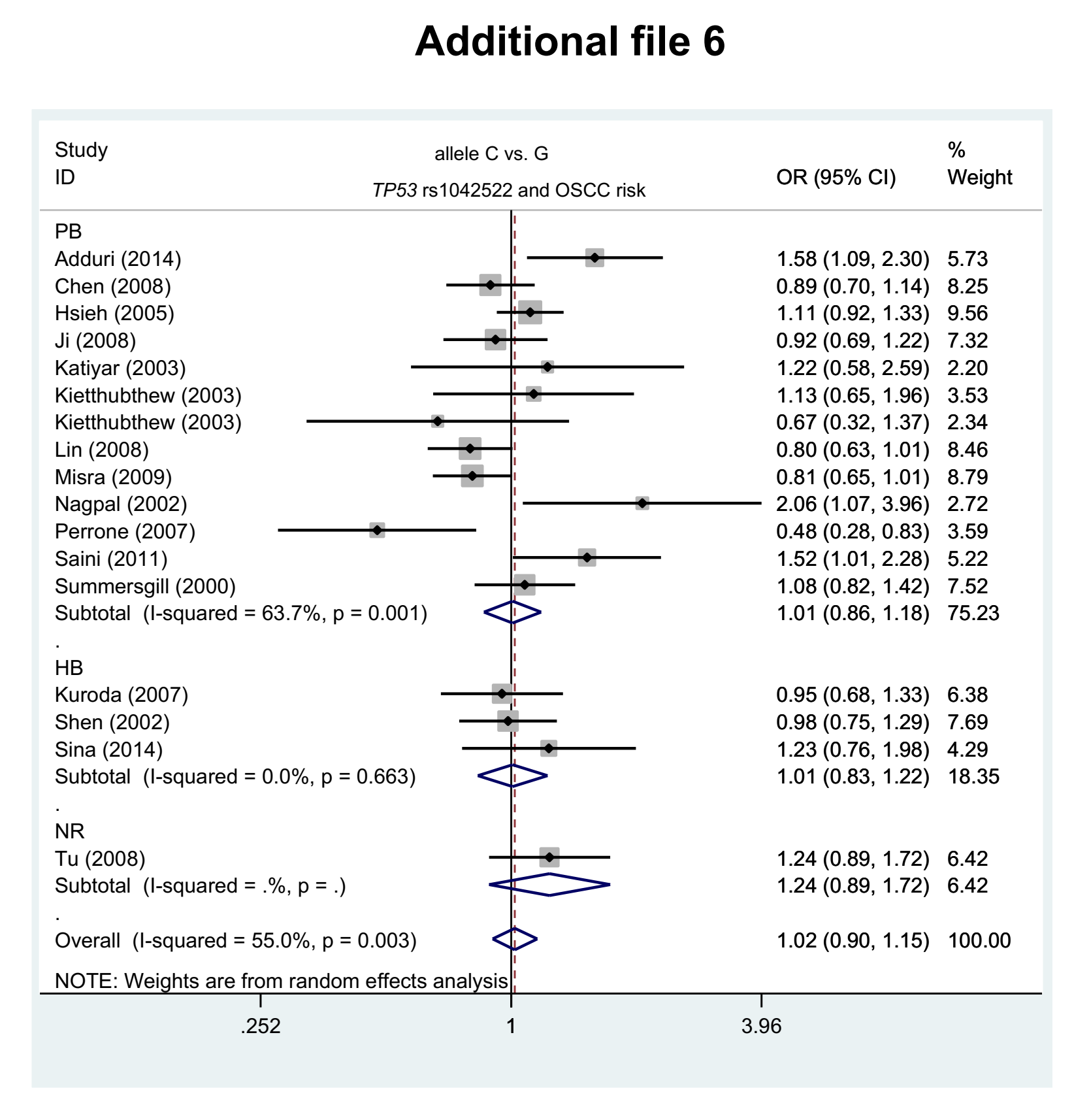

Supplement: Supplementary file 6 — Subgroup analysis (allele C vs. G) of OSCC by the control source. (TIF 1495 kb) [file 12903_2018_603_MOESM6_ESM.tif]

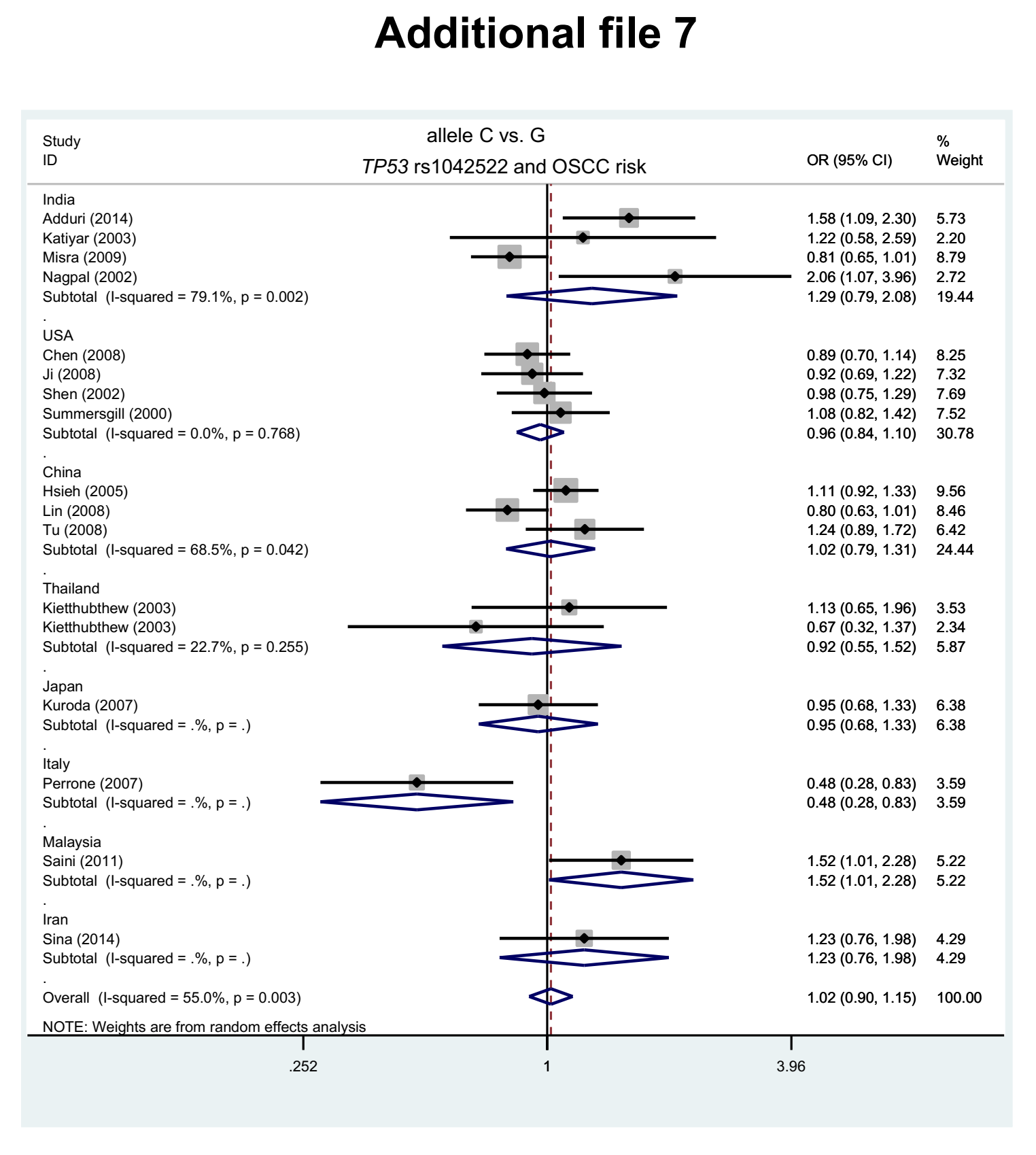

Supplement: Supplementary file 7 — Subgroup analysis (allele C vs. G) of OSCC by the location. (TIF 1307 kb) [file 12903_2018_603_MOESM7_ESM.tif]

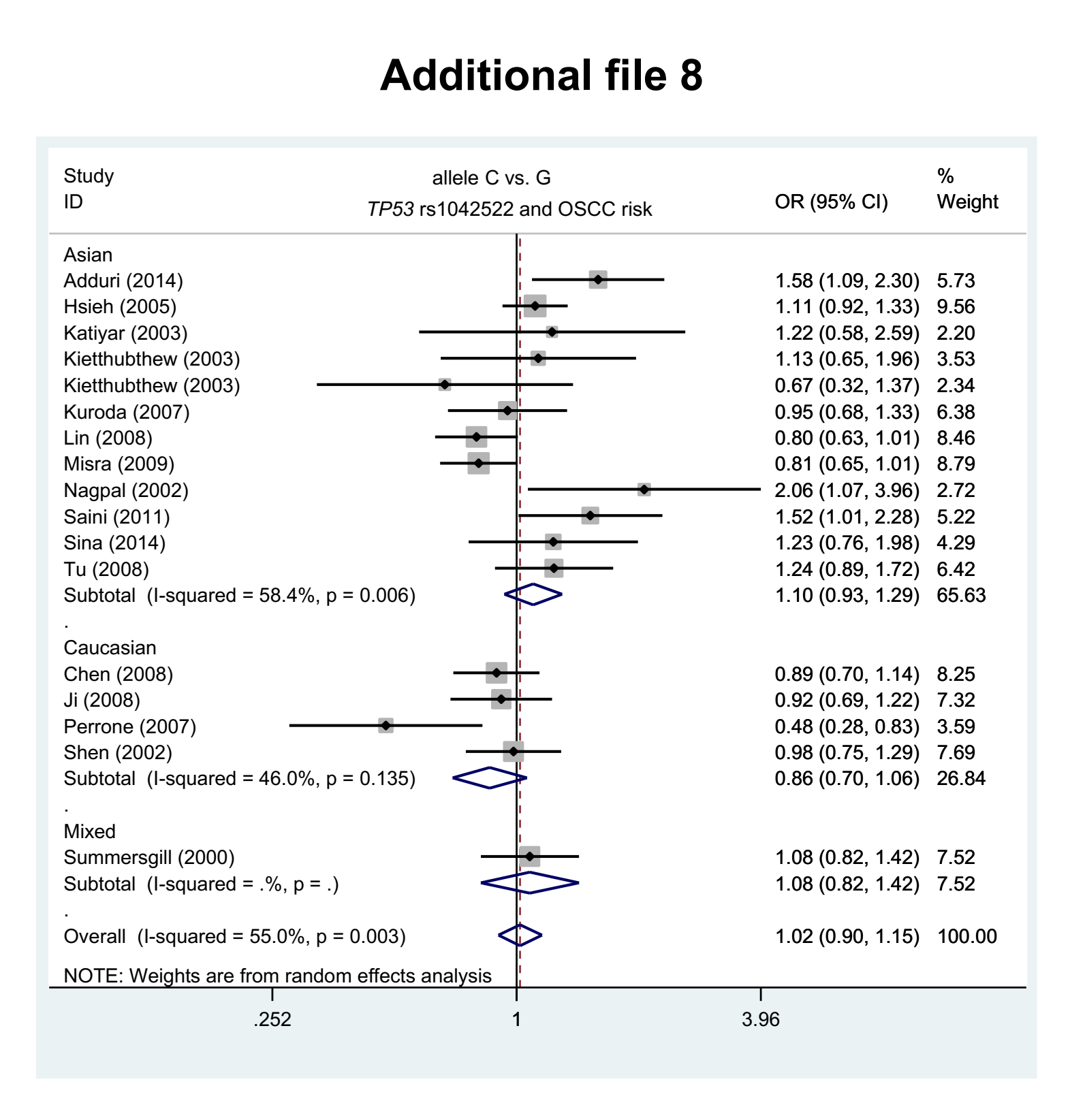

Supplement: Supplementary file 8 — Subgroup analysis (allele C vs. G) of OSCC by the ethnicity. (TIF 1362 kb) [file 12903_2018_603_MOESM8_ESM.tif]

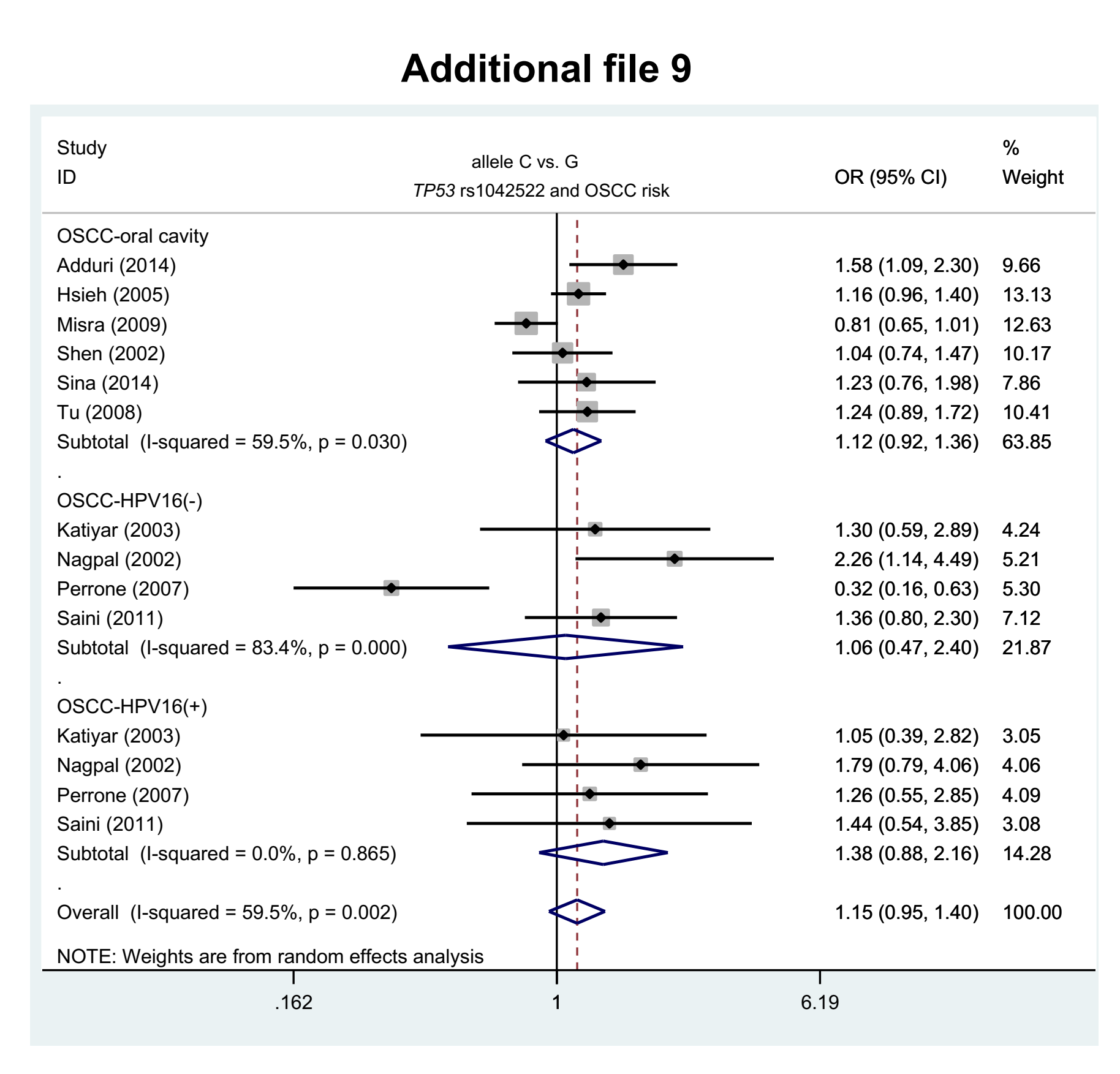

Supplement: Supplementary file 9 — Subgroup analysis (allele C vs. G) of OSCC by the disease type. (TIF 1498 kb) [file 12903_2018_603_MOESM9_ESM.tif]

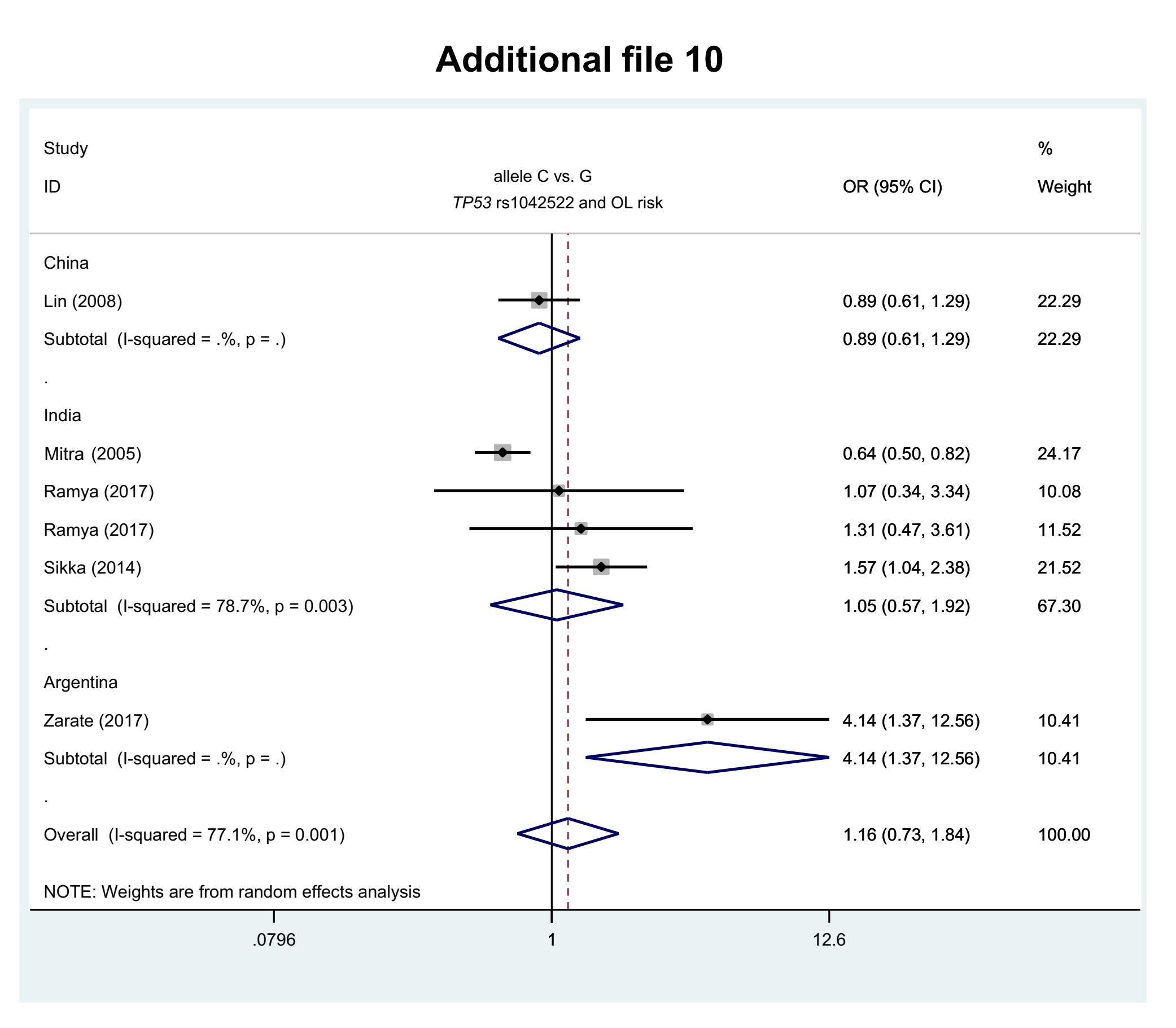

Supplement: Supplementary file 10 — Subgroup analysis (allele C vs. G) of OL by the location. (TIF 1225 kb) [file 12903_2018_603_MOESM10_ESM.tif]

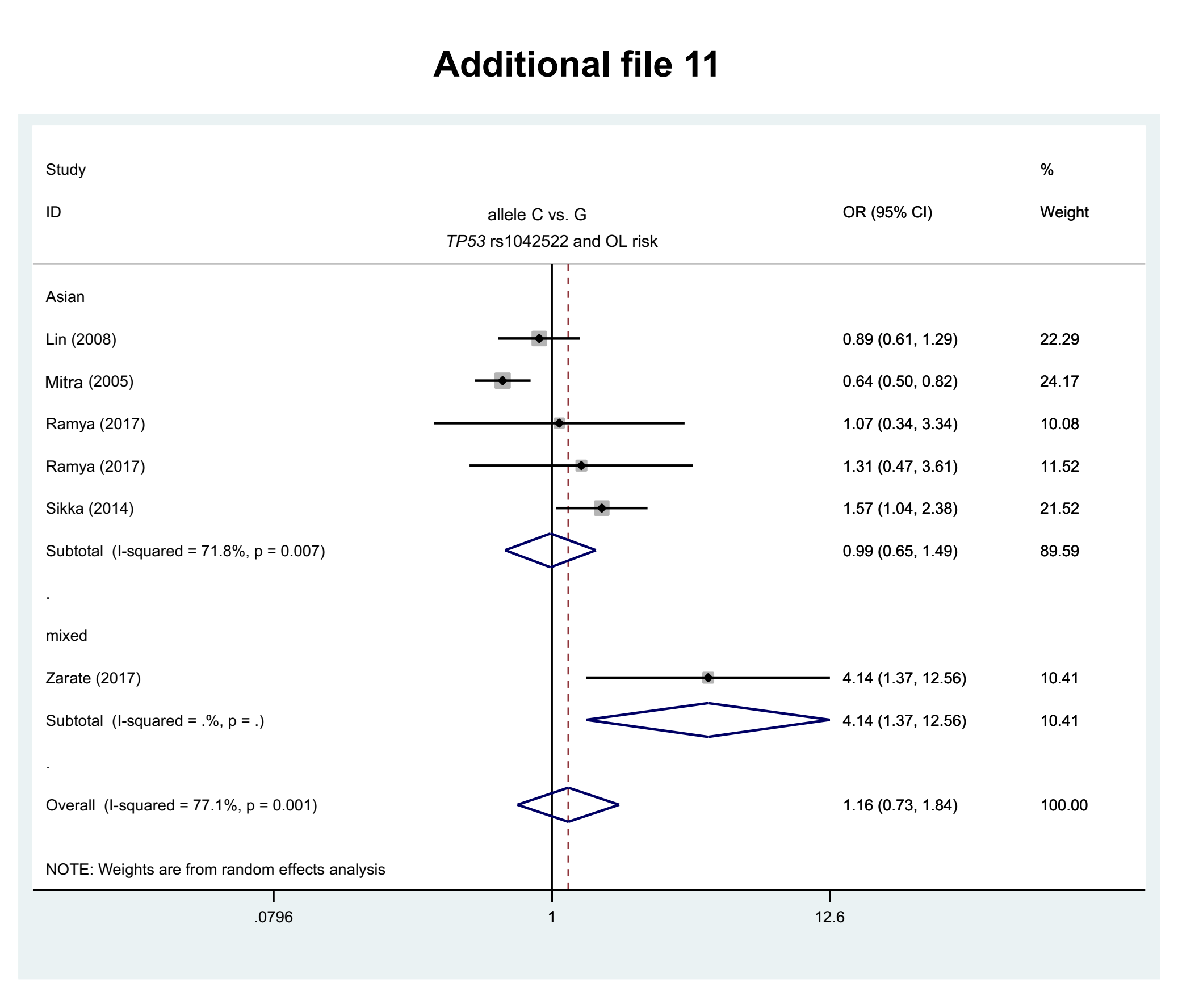

Supplement: Supplementary file 11 — Subgroup analysis (allele C vs. G) of OL by the ethnicity. (TIF 1092 kb) [file 12903_2018_603_MOESM11_ESM.tif]

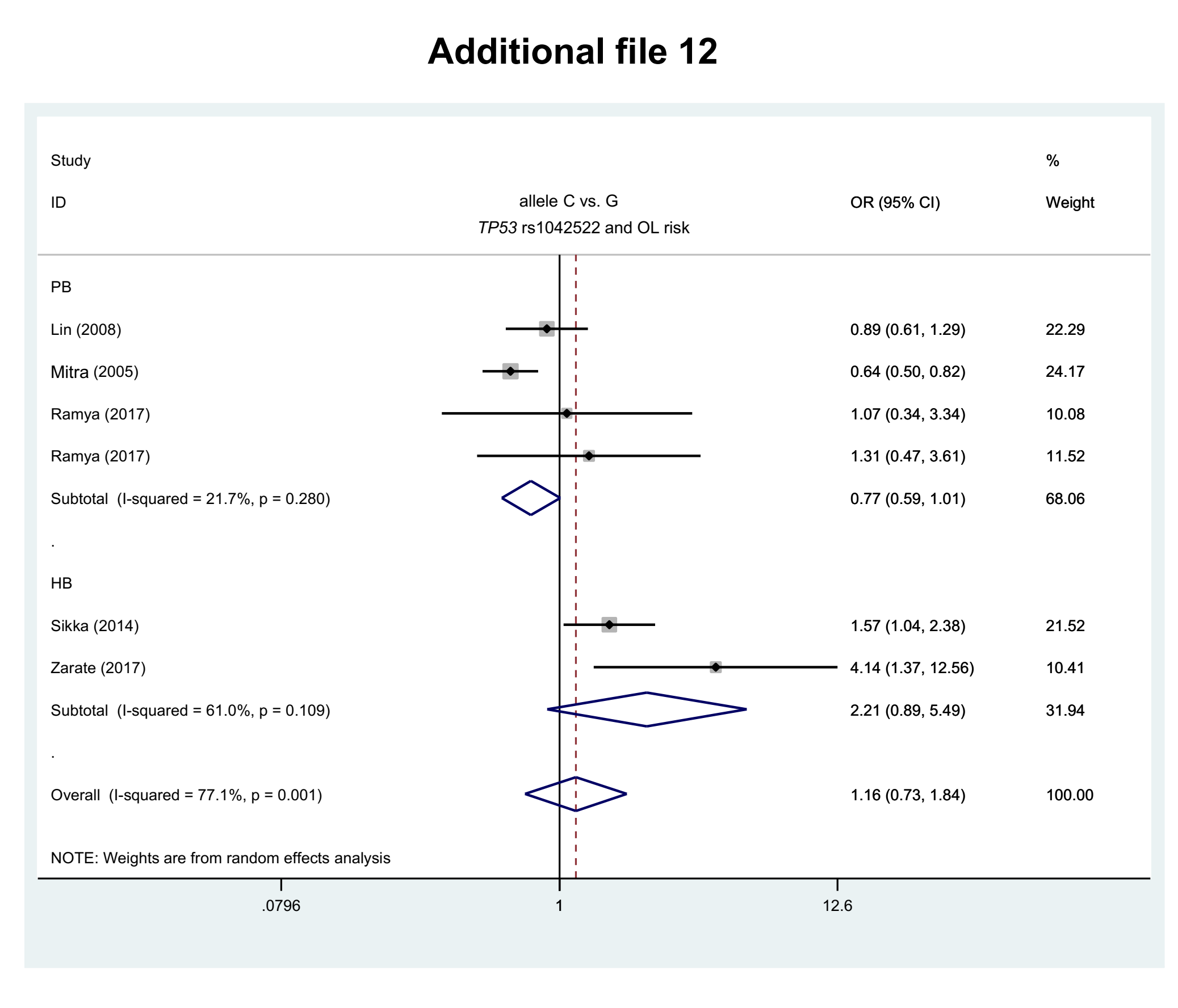

Supplement: Supplementary file 12 — Subgroup analysis (allele C vs. G) of OL by the control source. (TIF 1103 kb) [file 12903_2018_603_MOESM12_ESM.tif]

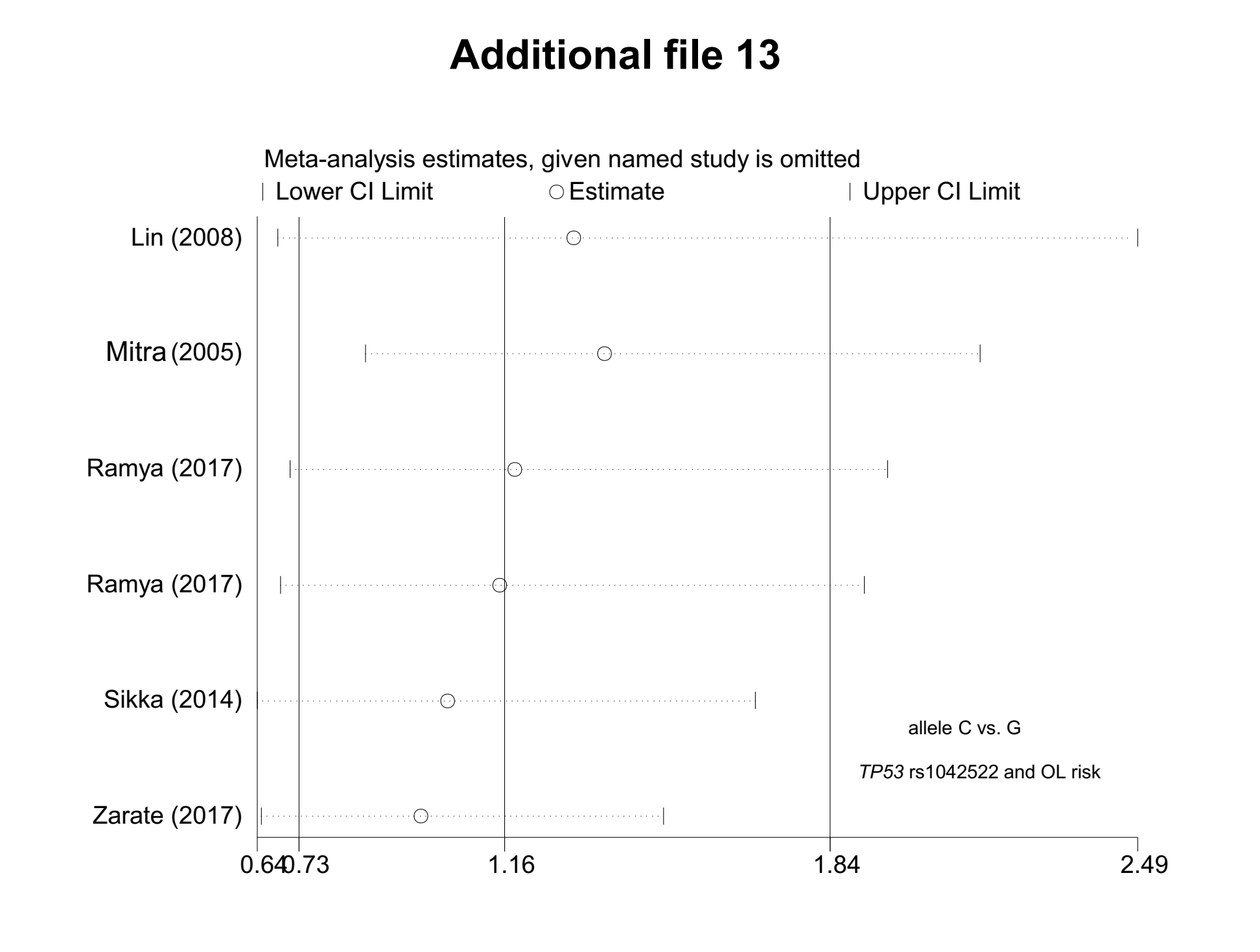

Supplement: Supplementary file 13 — Sensitivity analysis (allele C vs. G) of TP53 rs1042522 and OL risk. (TIF 358 kb) [file 12903_2018_603_MOESM13_ESM.tif]
